# The Humanistic Clinician

**DOI:** 10.1093/geroni/igaf122.1260

**Published:** 2025-12-31

**Authors:** James Powers

**Affiliations:** Vanderbilt University Medical Center, Nashville, Tennessee, United States

## Abstract

The Humanistic Clinician will invite audience participants to achieve new insights to enrich their appreciation for how we define the human condition including our capacity for empathy, care, compassion, vulnerability, resilience, and mutual support. The presentation will address changes in medicine, healthcare expectations of older adults, tensions between autonomy and interdependence surrounding healthcare decisions and guide-posts of humanistic practice.

